# Comparison of Immunological Characteristics of Mesenchymal Stem Cells from the Periodontal Ligament, Umbilical Cord, and Adipose Tissue

**DOI:** 10.1155/2018/8429042

**Published:** 2018-04-01

**Authors:** Jin-Hee Kim, Chris H. Jo, Hang-Rae Kim, Young-il Hwang

**Affiliations:** ^1^Department of Biomedical Laboratory Science, College of Health Science, Cheongju University, Chengju, Republic of Korea; ^2^Department of Orthopedic Surgery, SMG-SNU Boramae Medical Center, Seoul National University College of Medicine, Seoul, Republic of Korea; ^3^Department of Anatomy and Cell Biology, Seoul National University College of Medicine, Seoul, Republic of Korea

## Abstract

Mesenchymal stem cells (MSCs) are of therapeutic importance in the fields of regenerative medicine and immunological diseases. Accordingly, studies evaluating MSCs for clinical applications are increasing. In this study, we characterized MSCs from the periodontal ligament, umbilical cord (UC-MSCs), and adipose tissue, which were relatively easy to obtain with limited ethical concerns regarding their acquisition, and compared their immunological characteristics. Among MSCs isolated from the three different tissues, UC-MSCs grew the fastest *in vitro*. The three types of MSCs were shown to inhibit proliferation of activated peripheral blood mononuclear cells (PBMCs) to a similar degree, via the indoleamine 2,3-dioxygenase and cyclooxygenase-2 pathways. They were also shown to inhibit the proliferation of PBMCs using HLA-G, which was most prominent in UC-MSCs. Unlike the other two types of MSCs, UC-MSCs showed minimal expression of HLA-DR after activation, suggesting that they pose minimal risk of initiating an allogeneic immune response when administered *in vivo*. These characteristics, the ease of collection, and the minimal ethical concerns regarding their use suggest UC-MSCs to be suitable MSC therapeutic candidates.

## 1. Introduction

Mesenchymal stem cells (MSCs) are a population of adult stem cells found in most tissues that are characterized by their multipotency and self-renewal capacity. MSCs can differentiate into a wide range of specialized cells such as cardiomyocytes [1], neurons [2], and hepatocytes [3], in addition to adipocytes, chondrocytes, and osteocytes [4]. Thus, MSCs may have various applications in regenerative medicine [5, 6].

An important characteristic of MSCs is their immunosuppressive function. MSCs suppress the proliferation of activated T cells by secreting substances, such as indoleamine 2,3-dioxygenase (IDO) and prostaglandin E2 (PGE2) (reviewed in [7]), as well as inhibiting programmed death-ligand 1 (PD-L1) [8]. They also suppress the development of pro-inflammatory Th17 cells and stimulate regulatory T cells by secreting immunosuppressive cytokines including interleukin-6 (IL-6), IL-10, and hepatocyte growth factor (HGF) [7]. Owing to their immunomodulatory roles, MSCs have been explored as potential therapeutic agents for chronic inflammatory and immune diseases [9].

In fact, a severe case of graft-versus-host disease (GVHD) was treated using bone marrow-derived MSCs (BM-MSCs) from the patient's mother, which was the first clinical application of MSCs [10]. Thereafter, several MSC studies have been and are currently being applied in preclinical and clinical settings [11]. BM-MSCs have been used in most of these studies as the “golden standard” and as such have been well characterized.

However, there are several disadvantages to the therapeutic use of BM-MSCs. First, collection is invasive and efficiency is low compared with other sources of MSCs [12]. In addition, the number and differentiation potential of BM-MSCs decrease with aging [13].

Based on these characteristics, previous studies have explored other potential tissues for obtaining MSCs for clinical use, one of which is adipose tissue-derived MSCs (AD-MSCs). In the early 2000s, many investigators isolated and characterized multipotent stem cells from adipose tissue, which share similar characteristics to those of BM-MSCs [14, 15]. The method of AD-MSC collection is less invasive, and the yield is approximately 100- to 500-fold more than that of BM-MSCs [16].

Another potential source of MSCs is the umbilical cord (UC-MSCs). Collection of UC-MSCs is non-invasive, since they are obtained from medical waste discarded after childbirth. The isolation process is easy, and large amounts of MSCs can be collected prior to *in vitro* expansion [12]. Another advantage of UC-MSCs is that they grow faster (shorter doubling time) than BM-MSCs and are viable at higher passage numbers. In addition, UC-MSCs secrete higher concentrations of immunomodulatory substances, such as IL-10, IL-8, TGF-*β*2, and HGF [17].

Periodontal ligament stem cells (PDLSCs) were identified in 2004 [18] and can be isolated from discarded tissues following dental procedures. Their ease of isolation is advantageous in terms of accessibility, and there are minimal ethical concerns regarding their use.

Previous studies have compared MSCs from different tissue origins to BM-MSCs, the gold standard, to identify a better source of MSCs for clinical use. These studies suggested that AD- and/or UC-MSCs are good candidates to replace BM-MSCs.

However, limited comparisons have been made between PDLSCs and other MSCs. A previous study compared UC-MSCs, AD-MSCs, and PDLSCs [19] but did not investigate their immunological characteristics. Since MSCs are easily acquired from these three tissues, direct comparisons of these MSC populations are of importance.

In this study, we compared these three different sources of MSCs based on pluripotency, immunophenotype, and other immunological properties. We found that PDLSCs are comparable to UC- and AD-MSCs based on immunological characteristics. However, from a practical view, UC-MSCs are believed to be superior to the other two sources of MSCs.

## 2. Materials and Methods

### 2.1. Isolation and Preparation of MSCs

The MSCs used in this study were collected from three different tissues, including the umbilical cord, adipose tissue, and periodontal ligament. AD-MSCs and UC-MSCs were prepared as described previously [20, 21]. PDLSCs were isolated from human premolars, which were extracted from healthy adults who provided consent for orthodontic purposes. The teeth were digested with type I collagenase (Wako, Tokyo, Japan) and dispase (Gibco, Grand Island, NY, USA) in alpha-minimum essential medium (*α*-MEM; Gibco) at a concentration of 2 mg/mL for 1 hour at 37°C. The digested samples were passed through a 70 *μ*m strainer (Falcon®, Corning, NY, USA) to obtain single cells, which were incubated at 37°C and 5% CO_2_ in *α*-MEM supplemented with 10% fetal bovine serum (FBS; Gibco), 2 mM l-glutamine (Gibco), 100 U/mL penicillin (Gibco), and 100 mg/mL streptomycin (Gibco). Once the cultures reached 90% confluency, the cells were subcultured or stored in liquid nitrogen. Passage p4~8 cells were used in this study. Our study protocol was approved by the Institutional Review Board of Seoul National University (SNU-E-1107-017-368 and SNU-J-1511-005-715).

### 2.2. Immunophenotyping

Aliquots of 1 × 10^5^ MSCs were washed and suspended in PBS supplemented with 0.1% FBS. Cells were incubated with anti-CD34-PE, anti-CD45-PE, anti-CD73-PE, anti-CD90-FITC, anti-CD105-FITC, anti-CD80-FITC, anti-CD86-PE, anti-CD154-PE, anti-CD40-PE, anti-HLA-ABC-FITC, or anti-HLA-DR-FITC antibodies (all from BD, San Diego, CA, USA) for 30 minutes at 4°C and then washed twice in PBS containing 0.1% FBS. Cells were then resuspended in 200 *μ*L PBS containing 0.1% FBS and analyzed at 10,000 events per test using the FACSCalibur (BD Biosciences) or NovoCyte (ACEA Biosciences Inc., San Diego, CA, USA). Data were analyzed using NovoExpress 1.2.1 software.

### 2.3. MSC Differentiation

MSCs were induced to differentiate into adipocytes, osteoblasts, or chondrocytes using StemPro Differentiation Kits (Invitrogen, Carlsbad, CA, USA). The medium was changed twice per week. Differentiated adipocytes, osteoblasts, and chondrocytes were fixed in 4% paraformaldehyde solution on days 14, 28, and 21 of culture and stained with Red O (Sigma-Aldrich, St. Louis, MO, USA), Alizarin Red S (Sigma-Aldrich), or Alcian Blue (Sigma-Aldrich), respectively.

### 2.4. Estimation of the Proliferation Rate

MSC proliferation rates were assessed using the Cell Counting Kit-8 (CCK-8; Dojindo, Japan) according to the manufacturer's protocol with minor modifications. Briefly, 5 × 10^3^ cells/well were suspended in a 96-well plate in triplicate and maintained at 37°C and 5% CO_2_. Each well was incubated with 10 *μ*L CCK-8 solution for 2 hours, after which the absorbance at 450 nm was measured using the VICTOR3™ Multilabel Plate Reader (PerkinElmer, Waltham, MA, USA). Additionally, the cells were counted directly using the TC10™ automated cell counter (Bio-Rad Laboratories, Hercules, CA, USA) for further support of the results of the CCK-8 assay.

### 2.5. Peripheral Blood Mononuclear Cell (PBMC) Preparation and Activation

Human PBMCs were isolated from healthy donor blood samples (informed consent was provided) using the Ficoll-Paque PLUS density gradient. Collected PBMCs were seeded in 96-well plates at 2 × 10^5^/well containing 100 *μ*L RPMI 1640 Medium (Gibco) supplemented with penicillin/streptomycin and 10% FBS. Cells were stimulated with anti-CD3/CD28 antibody-coated beads (1 : 2 bead-to-cell ratio, Dynabeads® Human T-Activator CD3/CD28; Life Technologies, Oslo, Norway).

### 2.6. MSC Activation

MSCs were activated using two different methods: use of conditioned medium (CM) or IFN-*γ*. The CM was the supernatant from PBMC cultures, which was harvested 3 days following activation of the cells, as described above. IFN-*γ* (PeproTech, Rocky Hill, NJ, USA) was added to the culture medium at a final concentration of 1 or 10 ng/mL, as required. Subsequently, the cultures were incubated for 24 hours for transcriptional activation and 48 hours for translational activation.

### 2.7. Inhibition of PBMC Proliferation by MSCs

PBMCs were loaded with 5,6-carboxyfluorescein diacetate succinimidyl ester (CFSE) by incubating the cultures with medium containing 1 *μ*M CFSE for 30 minutes. CFSE-labeled PBMCs were seeded at 4 × 10^5^/well in a 24-well plate containing 500 *μ*L R10 medium (RPMI, 10% FBS, and 100 U/mL penicillin/streptomycin). Naive MSCs were added to the plate at an MSC : PBMC ratio of 1 : 25, 1 : 100, or 1 : 400. As necessary, 0.1 mM NS-398 (a COX-2 inhibitor; Sigma-Aldrich), 0.1 mM 1-methyl-l-tryptophan [1-MT] (an IDO inhibitor; Sigma-Aldrich), or a neutralizing antibody against HLA-G (clone 87G; EXBIO, Praha, Czech Republic) was added to the coculture. The inhibitor concentrations used were determined as the maximum concentration that maintained over 90% cell viability for both the MSC and PBMC cultures, as determined by the CCK-8 assay (data not shown). The concentration of neutralizing antibody used was based on previous reports. An IgG2a antibody (Diaclone, clone B-Z2) was used as an isotype control. T-cell proliferation was determined 3.5 days following coculture by flow cytometric analysis of CFSE fluorescence intensity.

### 2.8. RNA Isolation and Polymerase Chain Reaction (PCR)

Total RNA was purified from MSCs using the RNeasy Mini Kit (QIAGEN) according to the manufacturer's protocols. RNA samples were resuspended in diethyl pyrocarbonate-treated water, quantified, and stored at −80°C until use. cDNA was synthesized using the Transcriptor First Strand cDNA Synthesis Kit (Roche Applied Science, Basel, Switzerland), and PCR was performed using the PCR Master Mix Kit (Bioneer, Daejon, Korea). After 35 cycles, the PCR products were subjected to electrophoresis on a 2% agarose gel. Bands were visualized under UV light, and images were captured using the Multi-Image Light Cabinet. The densities of the bands were analyzed and normalized to the reference RPS18 using Quantity One software (Bio-Rad Laboratories). The primers used for RT-PCR are provided in Table 1.

### 2.9. Enzyme-Linked Immunosorbent Assay (ELISA)

The concentrations of TGF-*β*1, HGF, and soluble HLA-G in culture supernatants were measured by ELISA using Human TGF beta-1 Platinum ELISA Kit, HGF Human ELISA Kit (Thermo Fisher Scientific, Waltham, MA), and sHLA-G ELISA Kit (BioVendor, Brno, Czech Republic), respectively, following the manufacturer's instructions. Optical densities were measured at 450 nm using the VICTOR3 Multilabel Plate Reader (PerkinElmer, Waltham, MA, USA). Samples were from three independent experiments and were duplicated.

### 2.10. Statistical Analysis

Statistical analyses were performed using GraphPad Prism 6 software (GraphPad Software, La Jolla, CA, USA) or SPSS 11.5 for Windows. The data are presented as means ± SD. Comparisons between experimental groups were conducted using one-way analysis of variance (ANOVA) or Student's *t*-test. Tukey's post hoc analysis was then used to determine statistically significant differences between experimental groups, with *p* < 0.05 considered to indicate significance.

## 3. Results

### 3.1. Characterization of PDLSCs

**Figure 1:**
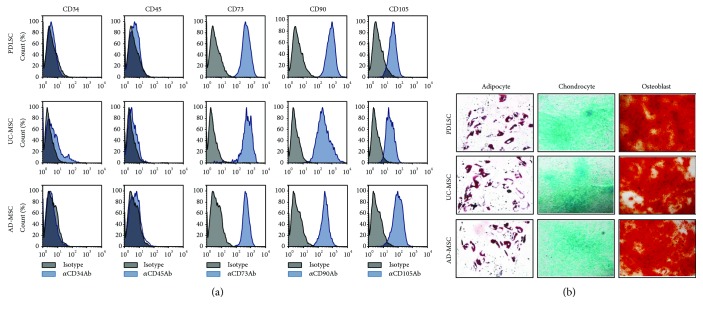
Characterization of human MSCs derived from the periodontal ligament (PDLSC), from the umbilical cord (UC-MSCs), and from the adipose tissue (AD-MSCs). (a) Cells were cultured for five to eight passages, harvested, labeled with antibodies against CD34, CD45, CD73, CD90, or CD105, and analyzed by flow cytometry. Black histograms indicate negative controls, and blue histograms represent PDLSCs stained with the indicated antibodies. (b) Cells were induced to differentiate into adipocytes, chondrocytes, or osteoblasts using the appropriate culture medium. Oil Red O, Safranin O, or Alizarin Red staining of adipocytes, chondrocytes, or osteoblasts was performed on days 14, 21, or 28 postinduction, respectively. All experiments were performed independently at least three times, and representative figures are shown.

Initially, we confirmed that the cells isolated from teeth were stem cells that met the defining characteristics outlined by the International Society for Cellular Therapy (ISCT) [4]. Cells that adhered to the culture plate and grew (data not shown) were positive for CD73, CD90, and CD105 and were negative for CD34, CD45, and HLA-DR (Figure 1(a)). These findings were similar to those for AD-MSCs [20] and UC-MSCs [21], despite minor differences in the mean fluorescence intensity (MFI) values for some markers. For example, CD90 was expressed most strongly in stem cells from teeth, while CD105 was highly expressed in AD-MSCs. Subsequently, we evaluated the ability of PDLSCs to differentiate into adipocytes, osteoblasts, and chondrocytes. In this experiment, passage 5 cells (p5) were used and successfully differentiated into the three cell types, which is consistent with UC-MSC and AD-MSC differentiation capacities (Figure 1(b)).

Collectively, our findings suggest that the cells isolated from the teeth specimens are MSCs (i.e., PDLSCs), as they met the stem cell criteria defined by the ISCT.

### 3.2. UC-MSCs Grew Faster than AD-MSCs and PDLSCs

**Figure 2:**
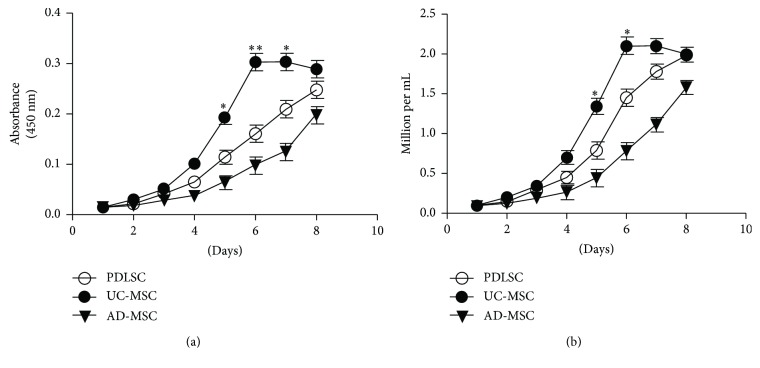
PDLSC, UC-MSC, and AD-MSC growth curves. Naive MSCs were seeded at 1 × 10^4^/well in a 24-well plate and cultured. The number of cells was counted every other day using the (a) Cell Counting Kit-8 (CCK-8) assay or (b) TC10 automated cell counter. The experiment was performed independently at least three times, and representative figures are shown. *p* values were obtained by ANOVA followed by Tukey's post hoc test. ^∗^ *p* < 0.05 and ^∗∗^ *p* < 0.001.

Considering up to 10 million MSCs/kg are required in clinical applications [22], rapid and extensive *in vitro* expansion of MSCs is important. Therefore, the PDLSC growth rate was compared with those of UC- and AD-MSCs, using p5 cells. The number of cells was estimated using the CCK-8 assay (Figure 2(a)) or counted directly using an automated cell counter (TC10; Bio-Rad Laboratories) (Figure 2(b)). The calculated doubling times for PDLSCs, UC-MSCs, and AD-MSCs were 42.7, 32.1, and 56.4 hours, respectively. The doubling time of PDLSCs was shorter than that of AD-MSCs but longer than that of UC-MSCs. In other words, UC-MSCs grew 1.33- and 1.75-fold faster than PDLSCs and AD-MSCs, respectively.

### 3.3. PDLSCs, UC-MSCs, and AD-MSCs Inhibited the Proliferation of Activated PBMCs

**Figure 3:**
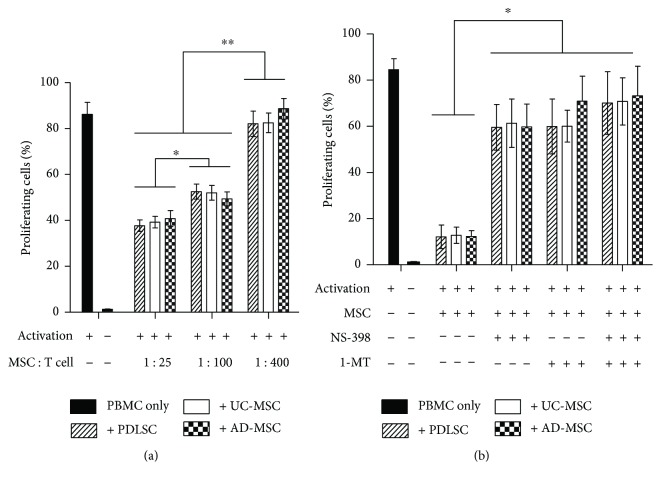
(a) Dose-dependent inhibition of activated PBMC proliferation by MSCs. PBMCs isolated from healthy donors were labeled with CFSE, stimulated with anti-CD3/anti-CD28 antibody-coated beads, and cocultured with naive MSCs at MSC : PBMC ratios of 1 : 25, 1 : 100, or 1 : 400 for 3.5 days. Proliferating cells were analyzed by flow cytometry, and the percentage of each experimental group is represented as a bar on the histogram. (b) Recovery of PBMC proliferation by inhibitors of IDO or COX-2. Identical experiments were performed as those in (a) at an MSC : PBMC ratio of 1 : 10 in the presence of the IDO inhibitor 1-methyl-l-tryptophan (1-MT, 0.1 mM), the COX-2 inhibitor (NS-398, 0.1 mM), or both. Flow cytometric profiles are shown in Supplementary Figure 2. All experiments were performed independently at least three times. The graph shows the means and standard errors of the mean (SEMs). *p* values were obtained by ANOVA followed by Tukey's post hoc test. ^∗^ *p* < 0.05 and ^∗∗^ *p* < 0.01.

MSCs are immunosuppressive considering that they inhibit T-cell proliferation *in vitro* [23]. To compare the immunosuppressive actions among the three sources of MSCs, CFSE-loaded PBMCs were activated with anti-CD3/CD28 antibody-coated beads in the presence of each type of MSC at varying MSC : T-cell ratios, after which the CFSE intensity was analyzed by flow cytometry. Our results showed that at greater numbers of MSCs (MSC : PBMC ratios ranging from 1 : 400 to 1 : 25), the proportion of divided cell fractions among PBMCs was decreased significantly (Figure 3(a) and Supplementary Figure 1A), regardless of the tissue source. The degree of suppression at specific cell ratios was similar among all three sources of MSCs.

T-cell suppression is mediated by MSC-secreted substances, such as IDO and PGE2 [24, 25]. To compare the mechanisms of immunosuppression among the MSC populations, we explored the expression of related enzymes by RT-PCR analysis and observed PCR bands for all three sources of MSCs after CM or IFN-*γ* treatment (Supplementary Figure 2). Next, we cocultured MSCs and PBMCs at a 1 : 10 ratio in the presence of NS-398 (COX-2 inhibitor) or 1-MT (IDO inhibitor) for 3.5 days (Figure 3(b) and Supplementary Figure 1B). Each inhibitor restored PBMC proliferation, the proportion of divided cell fraction being changed from 12% up to 60%. The magnitude of recovery was not significantly different among the different sources of MSCs (Figure 3(b)). Interestingly, the proliferation recovery achieved using NS-398 or 1-MT did not differ significantly in specific MSC experiments. Furthermore, when both inhibitors were added concomitantly, the effect was not additive or synergistic, with no substantial increase in recovery rate (Figure 3(b)).

**Figure 4:**
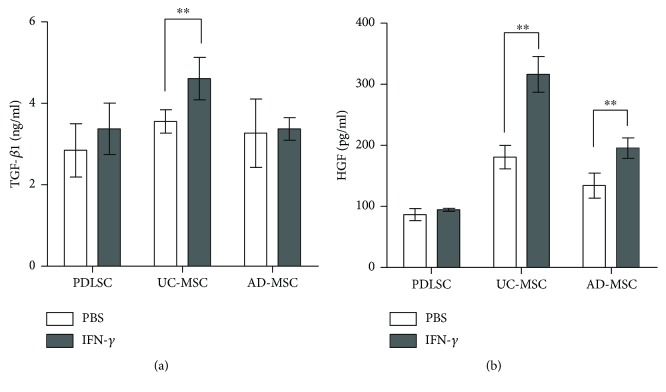
Secretion of immunosuppressive cytokines in MSCs. MSCs were cultured for 48 hours in the presence or absence of 10 ng/mL IFN-*γ*. Culture supernatants were subjected to ELISA for (a) TGF-*β*1 and (b) HGF using commercial ELISA kits. All experiments were performed three times, and duplicated samples from each experiment were measured. The graph shows the means and SEMs. *p* values were obtained by Kruskal-Wallis one-way analysis of variance. ^∗^ *p* < 0.05 and ^∗∗^ *p* < 0.01.

Apart from IDO and COX-2, MSCs secrete immunosuppressive cytokines such as TGF-*β*1 and HGF [17, 23]. Thus, we compared the secretion of these cytokine in the MSCs. All three types of MSCs constitutively secreted the cytokines, and UC-MSCs showed increased secretion of the cytokines upon activation with IFN-*γ* treatment (Figure 4).

### 3.4. HLA-G Was Expressed and Contributed to Inhibition of T-Cell Proliferation in All MSCs

**Figure 5:**
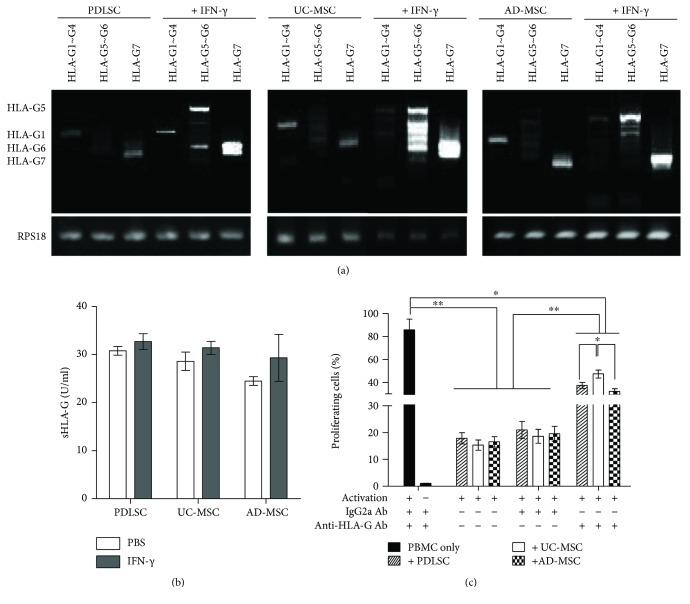
Expression of HLA-G following IFN-*γ* treatment. (a) RNAs were extracted from PDLSCs, UC-MSCs, and AD-MSCs prior to and following activation with 10 ng/mL IFN-*γ* for 24 hours, and RT-PCR analysis of the HLA-G subtypes was performed. The primers used are listed in Table 1. Ribosomal protein S18 (RPS18) was used as a reference gene. This experiment was performed three times, and a representative figure is shown. (b) Culture supernatants of each MSC, treated or nontreated with 10 ng/mL for 48 hours, were harvested and subjected for secretory HLA-Gs by ELISA. Samples were from three independent experiments and were duplicated. No statistical difference was observed among groups. (c) Inhibitory role of HLA-G on activated T-cell proliferation. A T-cell proliferation assay was performed using CFSE-loaded PBMCs. Cells were activated with anti-CD3 and anti-CD28 beads and cocultured with UC-MSCs, AD-MSCs, or PDLSCs at an MSC : PBMC ratio of 1 : 10 in the presence of PBS, neutralizing anti-HLA-G antibody (anti-HLA-G Ab), or isotype control antibody (IgG2a). All experiments were performed independently at least three times. The figure shows a graph depicting the means and SEMs for the experiments. Flow cytometric profiles are presented in Supplementary Figure 3.

In addition to IDO and COX-2, HLA-G, the nonclassical HLA class I molecule, is expressed in BM-MSCs [26] and UC-MSCs [27] and inhibits T-cell proliferation. However, the role of HLA-G in PDLSCs has yet to be explored. To explore its role, HLA-G expression was observed using RT-PCR and ELISA (Figure 5) in PDLSCs along with UC- and AD-MSCs, in the naive and activated states. Activation of MSCs was achieved through the addition of 10 ng/mL IFN-*γ* for 24 hours. In the naive state, all MSCs expressed HLA-G1 and -G7 (Figure 5(a)). However, when activated, PDLSCs expressed HLA-G1, -G5, and -G7, UC-MSCs expressed -G5, -G6, and -G7, and AD-MSCs expressed -G5 and -G7. The expression of HLA-Gs, in its secretory forms, was ascertained by ELISA (Figure 5(b)).

To determine whether the expressed HLA-G contributed to the inhibition of T-cell proliferation, CSFE-loaded T cells were activated and cultured in the presence of each MSC type in culture medium containing a neutralizing antibody against HLA-G (clone 87G) or mouse IgG2a antibody. Inhibition of T-cell proliferation was moderately recovered by 87G in the presence of all three MSC types; however, recovery of T-cell growth inhibition was greatest in the presence of UC-MSCs (Figure 5(c) and Supplementary Figure 3).

### 3.5. IFN-*γ* Treatment Increased HLA-ABC, as well as HLA-DR, Expression in AD-MSCs and PDLSCs, but Not in UC-MSCs

The inflammatory milieu has been shown to render MSCs immunogenic by upregulating HLA and other costimulatory molecules [28]. Since many clinical trials use allogeneic MSCs [22], it could be a problem if *in vivo* administration of MSCs stimulates an alloimmune response. Thus, we explored changes in MSC immunostimulatory surface molecules 2 days after activation with IFN-*γ* at a concentration of 1 ng/mL (a low dose), the reported maximum serum concentration in patients [29], or 10 ng/mL (a high dose, which is frequently used in *in vitro* studies).

**Figure 6:**
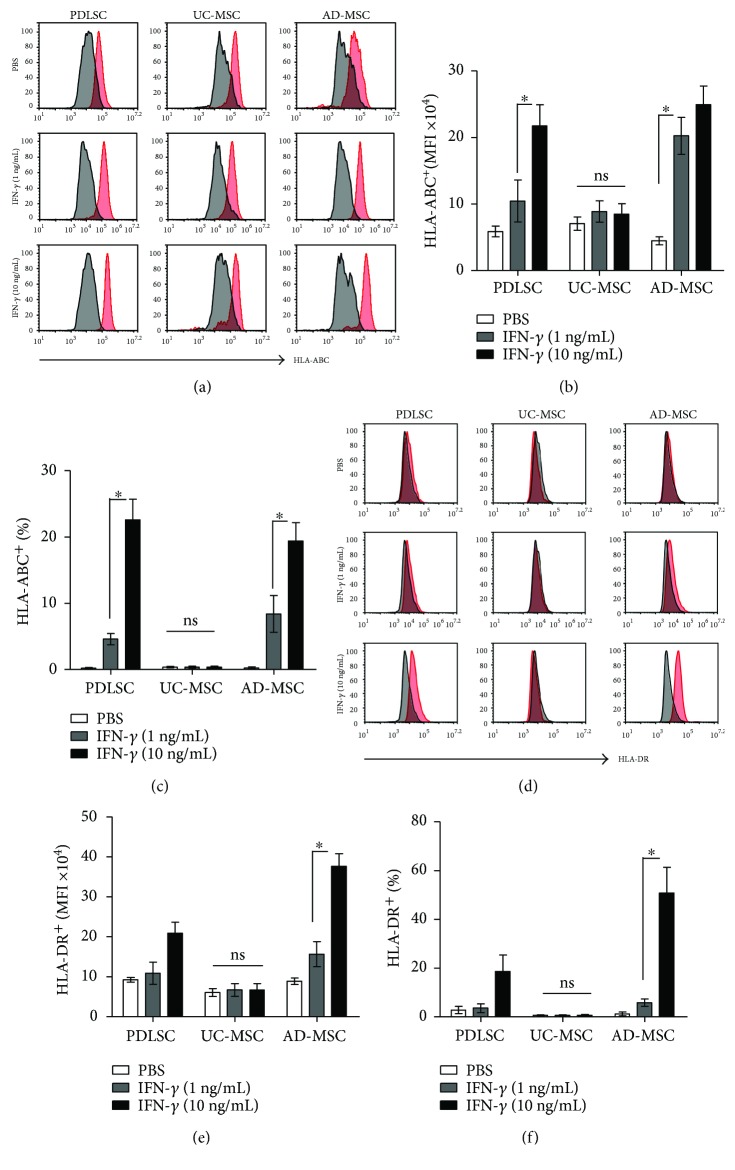
The effects of IFN-*γ* on the expression of HLA-ABC and HLA-DR. PDLSCs, UC-MSCs, and AD-MSCs at passage five were cultured without IFN-*γ* or with 1 or 10 ng/mL IFN-*γ* for 48 hours. Subsequently, cells were stained for (a) HLA-ABC and (d) HLA-DR, and the MFI values (b and e) and positive cell rates (c and f) were analyzed. Black histograms indicate negative controls, and red histograms represent MSCs stained with the indicated antibodies. All experiments were performed independently at least three times. The graph shows the means and SEMs. *p* values were obtained by ANOVA followed by Tukey's post hoc test. ^∗^ *p* < 0.05 and ns: not significant.

Expression of costimulatory molecules such as CD40, CD154, CD80, and CD86 did not change after IFN-*γ* treatment in any of the MSC types (data not shown). Each MSC type reacted differently in terms of HLA-ABC and HLA-DR expression. In PDLSCs, the MFI (Figures 6(a) and 6(b)) and frequency of HLA-ABC-positive cells (Figures 6(a) and 6(c)) increased after high-dose IFN-*γ* treatment, but no significant changes were observed after low-dose IFN-*γ* treatment. In addition, no discernable changes in HLA-DR expression were observed (Figures 6(d)–6(f)). In UC-MSCs, the expression of both HLA-ABC and HLA-DR was not affected by high-dose IFN-*γ*. In contrast, AD-MSCs responded to low-dose IFN-*γ*, showing an increased MFI for HLA-ABC, and to high-dose IFN-*γ*, showing increases in the MFI and frequencies of positive HLA-ABC and HLA-DR cells.

## 4. Discussion

In this study, we compared the characteristics of MSCs isolated from three different tissues (periodontal ligament, umbilical cord, and adipose tissue) that are easily obtainable with limited ethical concerns for clinical use. The MSC sources were compared in terms of differentiation potential, proliferation rate, immunomodulatory properties and mechanisms, secretion of additional immunosuppressive cytokines such as TGF-*β*1 and HGF, and potential immunogenicity. Among them, UC-MSCs had the shortest doubling time *in vitro*. Each of the MSCs used in this study showed comparable inhibitory effects on activated T-cell proliferation via IDO and COX-2 pathways. In addition, HLA-G-induced inhibitory effects were more prominent in UC-MSCs than in the other MSCs. We observed no increase in the surface expression of HLA molecules following activation with IFN-*γ* in UC-MSCs, which is opposite to the effects observed in the other two sources of MSCs (i.e., increased HLA-ABC and HLA-DR expression).

Clinical use of MSCs requires *in vitro* expandability. UC-MSCs showed the highest proliferation rate, which was three- to four-fold greater than that of AD-MSCs. These results are in agreement with previous findings from Amable et al. [17], who reported four-fold higher expandability of UC-MSCs compared with AD-MSCs. The growth rate of PDLSCs was intermediate to those of UC-MSCs and AD-MSCs. This sequence of expandability (i.e., UC-MSCs > PDLSCs > AD-MSCs) was observed previously by Trivanović et al. [19]. Meanwhile, Vangsness et al. [30] reviewed 1075 articles, with 29 articles included in the final analysis, and concluded that Wharton's jelly tissue provides the most promising yield of MSCs. Considering the various changes that stem cells undergo with aging, such as decreased self-renewal and responsiveness and genomic instability [31], it is expected that UC-MSCs from perinatal embryonic tissue have a higher proliferation rate compared with stem cells isolated from other tissues. Among MSCs isolated from different perinatal tissues, including the amnion, chorion, and umbilical cord, UC-MSCs exhibited the fastest doubling time [32]. Collectively, these findings suggest that UC-MSCs are more promising for clinical use than are AD-MSCs and PDLSCs in terms of *in vitro* expandability.

Ding et al. [33] showed that COX-2 expression and PGE2 secretion from PDLSC cells play a critical role in inhibition of activated T-cell proliferation, and this inhibition was recovered almost completely by indomethacin, a PGE2 inhibitor. The authors also observed no significant contribution by IL-10, TGF-*β*, or HGF to this mechanism. However, they did not discuss the role of IDO, which is one of the main factors involved in the inhibition of T-cell proliferation by MSCs [25]. These results differ from ours, since we observed only 60–70% recovery with the NS-398 COX-2 inhibitor. In addition, we observed the same level of recovery with the IDO inhibitor, 1-MT. A previous study explored the role of IDO in PDLSCs and found that IDO expression is stimulated by IFN-*γ* and is involved in the inhibition of T-cell proliferation [34]. Our RT-PCR results (Supplementary Figure 2) also showed upregulation of IDO expression in PDLSCs following incubation with conditioned media, as well as IFN-*γ*. Our findings suggest that IDO has important functions in PDLSCs.

Interestingly, the effects of 1-MT and NS-398 were neither synergistic nor additive. IDO exerts its effect on T-cell proliferation via accumulation of cytotoxic metabolites of tryptophan, such as kynurenine, 3-hydroxykynurenine, and 3-hydroxyanthranilic acid [35, 36], while COX-2 produces PGE2, which directly inhibits T-cell proliferation by increasing intracellular cAMP levels [37]. These two mechanisms appear to be complementary in MSC immunomodulation. Additionally, we showed that these effects occurred at a similar level in all MSCs evaluated in this study (Figure 3).

In our study, we found that HLA-G contributed to the inhibitory activity of the three MSC types against T-cell proliferation. HLA-G molecules are nonclassical HLA class I molecules that consist of seven isoforms: four membrane-bound (HLA-G1 to -G4) and three soluble (HLA-G5 to -G7) [38]. They were first described in maternal–fetal tolerance, and they exert immunosuppressive effects on various immune cells. HLA-G molecules inhibit cytotoxic T lymphocyte-mediated cytolysis [39] and T-cell proliferation [40]. These molecules also enhance the development of CD4^+^D25^high^FOXP3 regulatory T-cell development [26], induce the development of tolerogenic dendritic cells [41], and inhibit natural killer cell cytolytic functions in cells that secrete HLA-G and neighboring cells [26]. Since allogeneic MSCs may be immunogenic [42], HLA-G expression would be beneficial for the engraftment and survival of allogeneically administered MSCs. HLA-G has been shown to increase graft acceptance and decrease the risk of rejection in heart transplantations [43, 44].

In fact, several types of MSCs secrete HLA-Gs, including BM-MSCs [45], AD-MSCs [46], fetal liver-derived MSCs [46], and UC-MSCs [47]. In this study, we showed that PDLSCs also secrete HLA-Gs (G1, G5, and G7), similar to UC-MSCs and AD-MSCs. We further showed that HLA-G contributed to the T-cell inhibition properties of MSCs, as shown in the neutralizing antibody experiment (Figure 5). Important to note, recovery of T-cell inhibition by an anti-HLA-G neutralizing antibody was significantly higher for UC-MSCs than for the other MSC types. Considering that UC-MSCs are derived from fetal tissue, these cells are expected to use HLA-G more efficiently. Previous studies showed that UC-MSCs secrete HLA-G6, but not HLA-G5 [47], as observed in our study. Ding et al. [27] reported variable expression of HLA-G isoforms depending on the UC-MSC clone. Thus, it appears that the secreted isoform of HLA-G depends on the cell clone and tissue source of MSCs.

MSCs are considered immune-privileged because they express immunostimulatory molecules, such as CD40, CD156, CD80, CD86, and HLA molecules, at low levels [48]. However, several studies have provided contradictory results. When administered *in vivo*, MSCs localize to injured and inflamed areas [28], are activated by cytokines, and express surface molecules such as HLA-ABC/DR, which are target molecules for allorecognition, and thus initiate allogeneic immune responses [42]. Animal studies showed that alloimmune responses are mediated by MHC molecules [49, 50]. In human clinical trials, there is a lack of data suggesting that immune responses occur against allo-MSCs, since the procedures focus primarily on safety and only partially on efficacy, with no investigation of the potential for alloimmune responses [42, 51]. Thus, it is possible that alloimmune responses occur following donated allo-MSC treatment.

In this context, the upregulation of costimulatory and HLA molecules by activated MSCs is of concern when administering allogeneic MSCs. Thus, we explored the expression of immunostimulatory surface molecules presented on MSCs following their activation. We found that HLA-ABC and HLA-DR expression was increased in PDLSCs and AD-MSCs, but not in UC-MSCs, in response to a high dose of IFN-*γ*. These results are in agreement with previous PDLSC and UC-MSC findings [27, 52]. Collectively, these results suggest that UC-MSCs are more appropriate for allogeneic use than are AD-MSCs or PDLSCs. An important finding was that human UC-MSCs, in contrast to PBMCs, only slightly increase the serum alloresponsive IgG titer, pro-inflammatory cytokines, such as IFN-*γ* and TNF-*α*, and splenic T-cell activation when injected into humanized mice [53], supporting the low alloimmunogenicity of UC-MSCs.

In this study, we observed that PDLSCs shared similar characteristics with AD-MSCs and UC-MSCs. However, we did not compare the roles of secretory molecules, such as cytokines, chemokines, and growth factors, which are mediators of various MSC biological activities, among the MSC types. Further studies are required to explore this aspect of activity.

## 5. Conclusion

For MSC allogeneic clinical use, UC-MSCs, compared with PDLSCs and AD-MSCs, offer many advantageous characteristics including greater *in vitro* expandability, use of HLA-G, and minimal expression of HLA-DR upon activation. These characteristics, in addition to the harmless sample-acquisition procedure and minimal ethical concerns, suggest that UC-MSCs are the most promising candidates for allogeneic use.

## Figures and Tables

**Table 1:** List of primers used in this experiment.

Gene	Forward primer	Reverse primer	Product size (bases)
IDO^∗^	CGCTGTTGGAAATAGCTTC	CAGGACGTCAAAGCACTGAA	234
COX-2^∗^	TCCAAATGAGATTGTGGGAAAATTGCT	AGATCATCTCTGCCTGAGTATCTT	325
HLA-G1^∗∗^	AGGAGACACGGAACACCAAG	CCAGCAACGATACCCATGAT	685
HLA-G2/G4^∗∗^	AGGAGACACGGAACACCAAG	CCAGCAACGATACCCATGAT	409
HLA-G3^∗∗^	AGGAGACACGGAACACCAAG	CCAGCAACGATACCCATGAT	133
HLA-G5^∗∗^	AACCCTCTTCCTGCTGCTCT	GCCTCCATCTCCCTCCTTAC	895
HLA-G6^∗∗^	AACCCTCTTCCTGCTGCTCT	GCCTCCATCTCCCTCCTTAC	619
HLA-G7^∗∗^	ACCCTCTTCCTGCTGCTCT	TTACTCACTGGCCTCGCTCT	331
RPS18^∗∗∗^	GATGGGCGGCGGAAAATAG	GCGTGGATTCTGCATAATGGT	166

^∗^Primers from Kim et al. [20]. ^∗∗^Primers from Ding et al. [27]. ^∗∗∗^Primers from Samovski et al. [54].

## Supplementary Materials

Supplementary 1 Figure 1: flow cytometric profiles of the data presented in Figure 2. Panel A shows dose-dependent inhibition of PBMC proliferation by MSCs, and panel B shows the recovery of PBMC proliferation in the presence of an inhibitor of IDO or COX-2 or both inhibitors. The experiment was performed independently three times, and representative profiles are shown. W/o: without.

Supplementary 2 Figure 2: expression of COX-2 and IDO in MSCs. (A) Expression of COX-2 and IDO in MSCs after treatment with conditioned media (CM) for 1 day. RPS18 was used as a control gene. To obtain CM, PBMCs were stimulated with different T-cell stimulants for 3 days, including phorbol myristate acetate (10 ng/mL) and ionomycin (50 ng/mL) (PMA/I), lipopolysaccharide (LPS; 100 ng/mL), concanavalin A (ConA; 1 *μ*g/mL), or anti-CD3 and anti-CD28 antibody-coated beads (*α*CD3/*α*CD28 Abs). The primers used are listed in Table 1. (B) Expression of COX-2 and IDO in MSCs following treatment with IFN-*γ* for 1 day. (C) T-cell stimulants themselves did not induce expression of the enzymes. RPS18: ribosomal protein S18.

Supplementary 3 Figure 3: inhibitory role of HLA-G on activated T-cell proliferation. Flow cytometric profiles for the graph depicted in Figure 5(c). T-cell proliferation assay was performed using CFSE-loaded PBMCs in the presence of neutralizing anti-HLA-G antibody (*α*HLA-G Ab) or isotype control antibody (IgG2a). All experiments were performed independently at least three times.
