# Supplementary material for: Comparison of Immunological Characteristics of Mesenchymal Stem Cells from the Periodontal Ligament, Umbilical Cord, and Adipose Tissue
Source: Stem Cells Int. 2018 Apr 1;2018:8429042. doi: 10.1155/2018/8429042 (PMC5901833; doi:10.1155/2018/8429042)
Supplement: Supplementary 1 — Figure 1: flow cytometric profiles of the data presented in Figure 2. Panel A shows dose-dependent inhibition of PBMC proliferation by MSCs, and panel B shows the recovery of PBMC proliferation in the presence of an inhibitor of IDO or COX-2 or both inhibitors. The experiment was performed independently three times, and representative profiles are shown. W/o: without. [file 8429042.f1.pptx]

## Slide 1
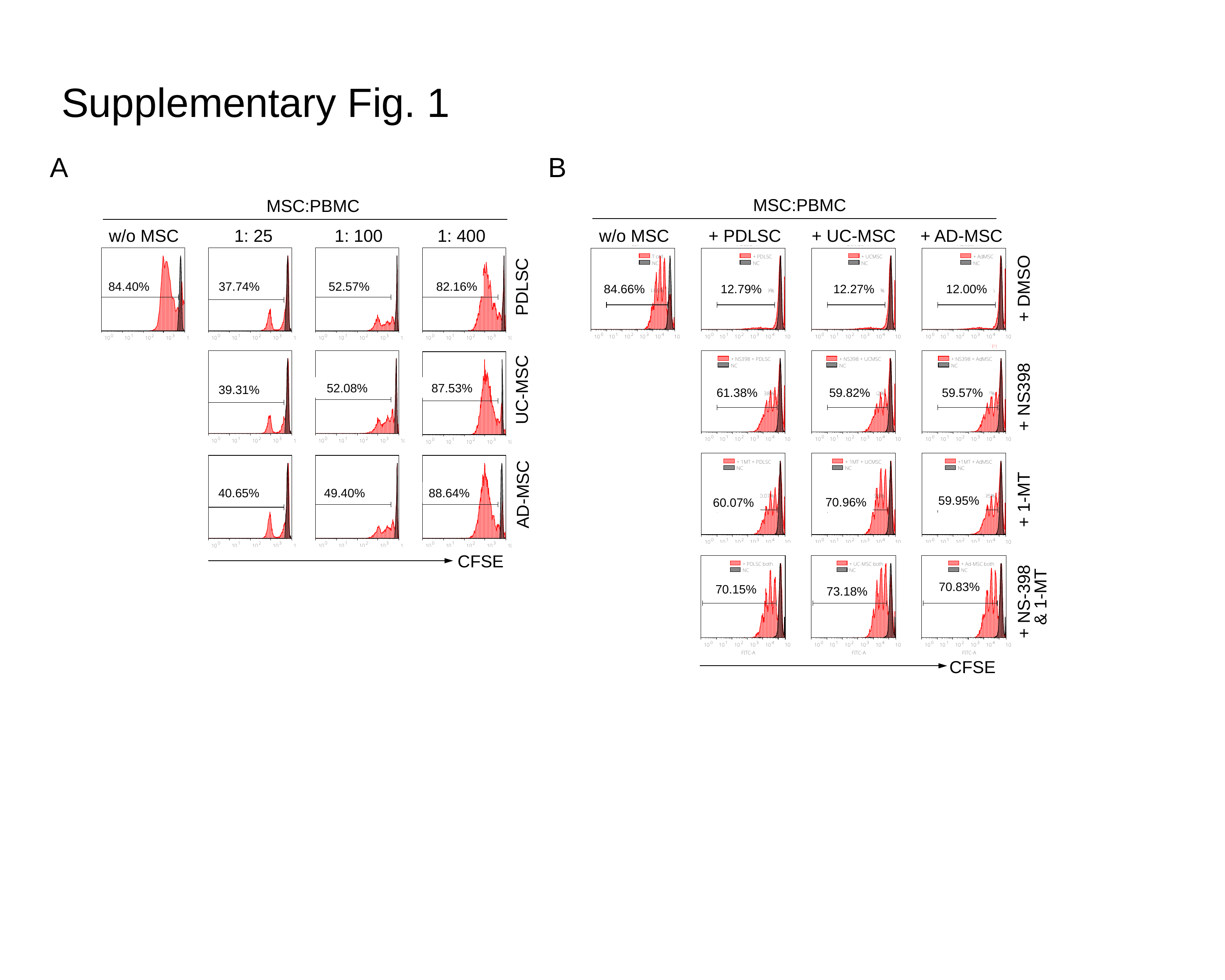

Supplementary Fig. 1
A
B
MSC:PBMC
w/o MSC
+ PDLSC
+ UC-MSC
+ AD-MSC
+ DMSO
84.66%
12.79%
12.27%
12.00%
61.38%
59.82%
59.57%
61.38%
59.82%
59.57%
+ NS398
+ 1-MT
59.95%
70.96%
60.07%
70.83%
70.15%
73.18%
+ NS-398
 & 1-MT
CFSE
MSC:PBMC
1: 25
1: 100
1: 400
w/o MSC
84.40%
37.74%
52.57%
82.16%
PDLSC
39.31%
52.00%
52.08%
82.53%
87.53%
39.31%
UC-MSC
40.65%
49.40%
88.64%
AD-MSC
CFSE
