# Supplementary material for: Comparison of Immunological Characteristics of Mesenchymal Stem Cells from the Periodontal Ligament, Umbilical Cord, and Adipose Tissue
Source: Stem Cells Int. 2018 Apr 1;2018:8429042. doi: 10.1155/2018/8429042 (PMC5901833; doi:10.1155/2018/8429042)
Supplement: Supplementary 2 — Figure 2: expression of COX-2 and IDO in MSCs. (A) Expression of COX-2 and IDO in MSCs after treatment with conditioned media (CM) for 1 day. RPS18 was used as a control gene. To obtain CM, PBMCs were stimulated with different T-cell stimulants for 3 days, including phorbol myristate acetate (10 ng/mL) and ionomycin (50 ng/mL) (PMA/I), lipopolysaccharide (LPS; 100 ng/mL), concanavalin A (ConA; 1 μg/mL), or anti-CD3 and anti-CD28 antibody-coated beads (αCD3/αCD28 Abs). The primers used are listed in Table 1. (B) Expression of COX-2 and IDO in MSCs following treatment with IFN-γ for 1 day. (C) T-cell stimulants themselves did not induce expression of the enzymes. RPS18: ribosomal protein S18. [file 8429042.f2.pptx]

## Slide 1
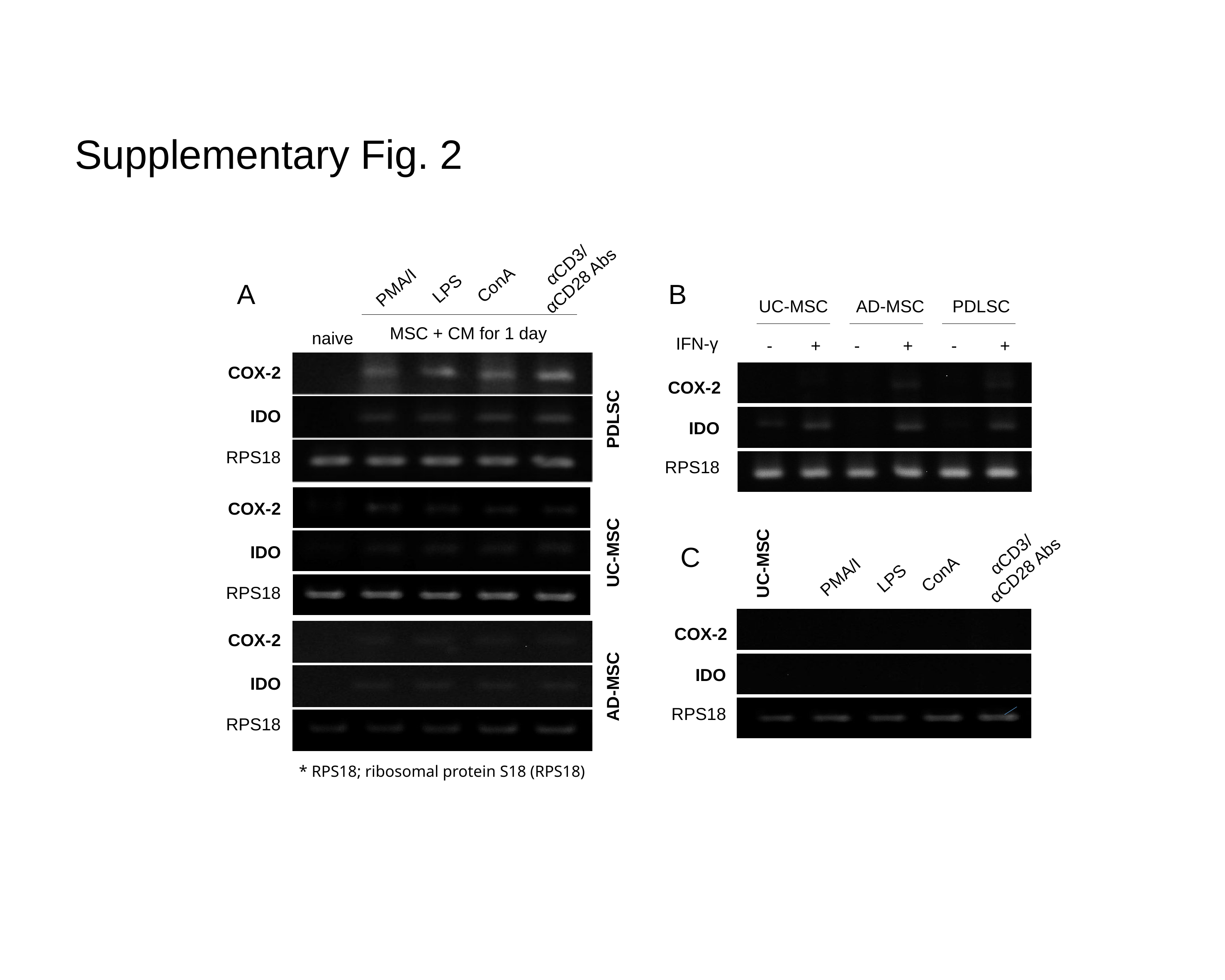

Supplementary Fig. 2
αCD3/
αCD28 Abs
ConA
PMA/I
LPS
MSC + CM for 1 day
naive
COX-2
IDO
PDLSC
RPS18
COX-2
IDO
UC-MSC
RPS18
COX-2
IDO
AD-MSC
RPS18
* RPS18; ribosomal protein S18 (RPS18)
A
B
UC-MSC
AD-MSC
PDLSC
IFN-γ
- + - + - +
COX-2
IDO
RPS18
αCD3/
αCD28 Abs
UC-MSC
ConA
PMA/I
LPS
COX-2
IDO
RPS18
C
