# Supplementary figures and images for: Comparison of Immunological Characteristics of Mesenchymal Stem Cells from the Periodontal Ligament, Umbilical Cord, and Adipose Tissue
Source: Stem Cells Int. 2018 Apr 1;2018:8429042. doi: 10.1155/2018/8429042 (PMC5901833; doi:10.1155/2018/8429042)

## Slide 1
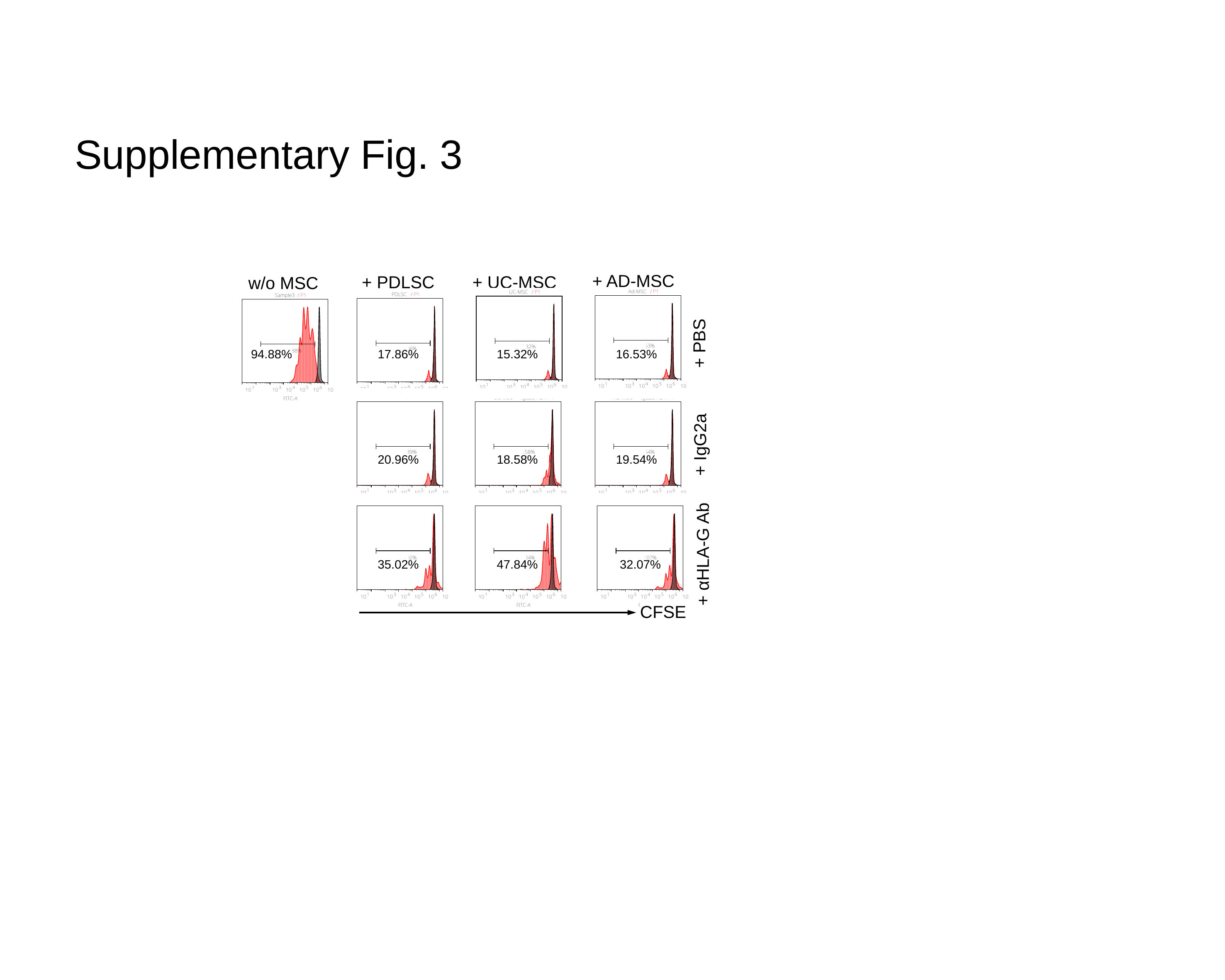

Supplementary Fig. 3
+ AD-MSC
w/o MSC
+ PDLSC
+ UC-MSC
94.88%
17.86%
15.32%
16.53%
20.96%
18.58%
19.54%
35.02%
47.84%
32.07%
+ PBS
+ IgG2a
+ αHLA-G Ab
CFSE

Supplement: Supplementary 3 — Figure 3: inhibitory role of HLA-G on activated T-cell proliferation. Flow cytometric profiles for the graph depicted in Figure 5(c). T-cell proliferation assay was performed using CFSE-loaded PBMCs in the presence of neutralizing anti-HLA-G antibody (αHLA-G Ab) or isotype control antibody (IgG2a). All experiments were performed independently at least three times. [file 8429042.f3.pptx]
